# Effects of acute cigarette smoke exposure on macrophage kinetics and release of tumour necrosis factor α in rats

**DOI:** 10.1155/S0962935193000171

**Published:** 1993

**Authors:** G. P. Pessina, L. Paulesu, F. Corradeschi, E. Luzzi, A. Di Stefano, M. Tanzini, G. Matteucci, V. Bocci

**Affiliations:** 1 1Institute of General Physiology University of Siena via Laterina 8 Siena 53100 Italy; 2 Department of Molecular Biology University of Siena Siena 53100 Italy; 3 Biocine-Sclavo S.p.A via Fiorentina Siena 53100 Italy

## Abstract

Some biological parameters before and after an acute episode of cigarette smoking in rats have been evaluated. The carboxyhaemoglobin levels depended either on the number of cigarettes, or on the time of exposure to cigarette smoke and returned to pre-smoking values in about 2 h. The evaluation of the kinetics of alveolar and peritoneal macrophages in rats after a smoking session of three cigarettes within an hour, indicated that alveolar macrophages in the bronchoalveolar lavage fluid significantly increased 8 h after the smoking, whereas the number of peritoneal macrophages remained practically constant. The incubation of these cells for various times at 37^°^C in a humidified atmosphere, resulted in a spontaneous release, 24 h thereafter, of variable amounts of tumour necrosis factor α (TNFα), which remained practically constant during the following days. Neither alveolar macrophages of control rats, nor peritoneal macrophages of both control and smoking rats were able to release TNFα. Moreover, after lipopolysaccharide induction of alveolar macrophages of both control and smoking rats, an increased release of TNFα was observed, indicating that these cells were in an active state.


Mediators of Inflammation, 2, 119–122 (1993)

### Effects of acute cigarette smoke exposure on macrophage kinetics and release of tumour necrosis factor α in rats

G. P. Pessina,[1] L. Paulesu,[1] F Corradeschi,[1] E. Luzzi,[1] A. Di Stefano,[2] M Tanzini,[1] G. Matteucci[3] and V. Bocci[1,CA]

[1]Institute of General Physiology, University of Siena, via Laterina 8, 53100 Siena, Italy; [2]Department of Molecular Biology, University of Siena, 53100 Siena, Italy; [3]Biocine-Sclavo S.p.A., via Fiorentina, 53100 Siena, Italy

[CA] Corresponding Author

**SOME biological parameters before and after an acute episode of cigarette smoking in rats have been evaluated. The carboxyhaemoglobin levels depended either on the number of cigarettes, or on the time of exposure to cigarette smoke and returned to pre-smoking values in about 2 h. The evaluation of the kinetics of alveolar and peritoneal macrophages in rats after a smoking session of three cigarettes within an hour, indicated that alveolar macrophages in the bronchoalveolar lavage fluid significantly increased 8 h after the smoking, whereas the number of peritoneal macrophages remained practically constant. The incubation of these cells for various times at 37°C in a humidified atmosphere, resulted in a spontaneous release, 24 h thereafter, of variable amounts of tumour necrosis factor α (TNFα), which remained practically constant during the following days. Neither alveolar macrophages of control rats, nor peritoneal macrophages of both control and smoking rats were able to release TNFα. Moreover, after lipopolysaccharide induction of alveolar macrophages of both control and smoking rats, an increased release of TNFα was observed, indicating that these cells were in an active state.**

**Key words:** Alveolar macrophages, Cigarette smoke, LPS induction, TNFα

### Introduction

Cigarette smoking is a useful human model of chronic inflammation[1] and it is well known that inflammation may contribute to the destruction and remodelling of normal lung architecture. Cigarette smoking, exposing the surface of the lower respiratory tract to more than 4 000 chemical constituents,[2] has been associated with an inflammatory process.[3] Moreover, inflammation leads to an emigration, either of monocytes or polymorphonuclear cells, from the vasculature to the tissue. Alveolar macrophages, which are derived from blood monocytes, exert their inflammatory activities at the various sites either directly, or through the action of tumour necrosis factor (TNF) and other immunological mediators.[4] TNFα is chemotactic for monocytes and polymorphonucleate cells (PMN), stimulates phagocytosis, causes adherence to endothelium and production of oxygen-derived metabolites, induces procoagulant activities in cultured human endothelial cells, and activates macrophages resulting in interleukin-1 and prostaglandin E2 production.[4–6] Thus the locally regulated generation of TNFα at sites of injury represents an important autocrine–paracrine control mechanism which operates in acute inflammatory response.[4]

Evaluation of the acute effect of cigarette smoking in human non-smokers is uncertain because they do not inhale cigarette smoke deeply.[7] For this reason research has been carried out to study either the acute effect of cigarette smoking in human smokers,[7] or in small animals,[8] or the effect of cigarette smoke in blood cells *in vitro*.[9,10] Assuming that the activation of macrophages is an important event for the pleiotropic effects of these cells, the present study was undertaken to investigate some biological parameters after acute tobacco smoking and the ability of cigarette smoke to activate rat alveolar macrophages, thus causing the release of TNFα. We also examined whether the release of TNFα by alveolar macrophages of rats exposed to an episode of acute, passive cigarette smoking, would be influenced by the presence of lipopolysaccharide (LPS).

### Materials and Methods

*Animals:* Outbred Wistar male rats (Charles River) of about 280 g body weight were used throughout the experiments. Groups of six rats were each lodged in the smoke chamber for various periods of time as indicated in Table 1. Smoke was generated in the smoke apparatus using a varied number of cigarettes without filter (Camel). This particular brand was chosen only because it is an internationally known brand of cigarettes. Immediately after smoking (0 h) and after 2, 4, 8 and 24 h, animals were sacrificed by an overdose of nembutal (Serva, Heidelberg). Blood was withdrawn from the femoral vein with an insulin type syringe containing

© 1993 Rapid Communications of Oxford Ltd

**Table 1.** Correlation between number of cigarettes, smoking period and COHb blood levels in rats measured immediately after the smoking session

| Smoking (h) | Cigarettes (*n*) | Rats (*n*) | COHb (%) |
|---|---|---|---|
| 2 | 1 | 6 | 17.1 ± 2.2 |
| 1 | 3 | 8 | 16.4 ± 3.6 |
| 1 | 1 | 6 | 9 ± 2.5 |
| 0 | 0 | 4 | −1 ± 1.1 |

10 IU/ml of heparin (Liquemin, Roche), for the measurement of carboxyhaemoglobin (COHb), by reading the absorbance at 276.5 nm in a spectrophotometer (Spectra Comp.601, Carlo Erba). Afterwards, 10 ml of sterile Hanks' balanced solution, pH 7.4, containing 5 IU/ml heparin was injected into the peritoneal cavity, the abdomen was gently massaged and then about 7 ± 1 ml of peritoneal fluid (PF) was aspirated for the collection of peritoneal macrophages.

Bronchoalveolar lavage was performed in the rats through a catheter inserted into the trachea with heparinized (10 IU/ml) sterile Hanks' solution. Five washes of 10 ml each were carried out in rapid succession and the lavage fluids were pooled. Recovery was about 90%. Lavage fluid was then centrifuged at 300 × ***g*** for 10 min at 4°C and the sedimented cells shown to be macrophages (more than 95%) by nonspecific esterase staining. Separated macrophages were tested for viability by the Trypan blue exclusion method and more than 95% of cells were found to be viable. The number of macrophages was evaluated by at least three countings in a Bürker chamber.

Control animals (air-sham exposed rats) were subjected to the same procedure except that the chamber was insufflated with air only.

*Cell culture:* For the experiments evaluating the eventual production of TNFα, cells from BAL and PF, collected immediately after (0 h) and 4, 8 and 24 h after the smoking session of three cigarettes for 1 h, were diluted to the desired final concentration ($10^6$ cells/ml) with RPMI 1640 containing 10% heat-inactivated foetal calf serum (FCS), 2 mM glutamine, 100 U/ml penicillin G and 100 μg/ml streptomycin. One hundred microlitres of the cell suspensions (about $10^5$ cells) were added to each well in 96-well culture plates (Costar) and were incubated for 24, 48 and 72 h at 37°C in a humidified atmosphere (95% air/5% $CO_2$). A second batch of cells, collected 0, 8 and 24 h after the smoking session and layered as above, was challenged with 1 μg/ml bacterial lipopolysaccharide (LPS) from *Escherichia coli* (Sigma) and incubated for 24 h at 37°C. Then the plates were centrifuged and supernatants were stored at −80°C until TNFα was determined.

Sediments, after two washings with normal saline, were dissolved in 0.1 N NaOH (0.1 ml per well, 12 h at room temperature) and proteins were measured by the method of Lowry *et al.*[11] with some modifications.

*TNFα determination:* TNF activity was determined by the method of Ruff and Gifford[12] with some modifications. Briefly, L929 cells were seeded at a density of 4 × $10^4$ cells per well in 96-well culture dishes in 100 μl of Eagle's MEM supplemented with 10% FCS and antibiotics. After 4 h incubation at 37°C in a humidified atmosphere (95% air/5% $CO_2$), two-fold serial dilutions of the samples in 2 μg/ml of actinomycin D (Serva), were prepared in separate 96-well culture dishes. Then 100 μl of each dilution was transferred into the corresponding well and the plates were further incubated for 18 h at 37°C in the humidified atmosphere. Supernatants were then removed and cells stained with 0.1% crystal violet. After drying, 100 μl of 33% acetic acid was added to each well to dissolve the dye. Plates were finally read at 540 nm on a Titertek Multiskan microElisa reader. Units of TNF activity were defined as the reciprocal of the dilution causing 50% of maximum cytotoxicity. Human Recombinant TNFα (Biogen BASF/Knoll) with a specific activity of 1.5 × $10^7$ units/mg protein was used as internal standard.

*Statistical analysis:* TNFα activity is reported as units/mg protein, 1 mg protein corresponding to 1.7 ± 0.5 × $10^7$ cells. Evaluation of experimental data was performed using either Mann–Whitney's *U* test or two-tailed Student's *t*-test with $p \leq 0.05$ as the minimal level of significance.

### Results

Because of the lack of a readily and simply measurable marker of environmental tobacco smoke, COHb levels were assessed as a reliable indicator of the biological effects of cigarette smoking. Normally in non-smokers, the average of basal COHb levels was −1 ± 1.1 and, as shown in Table 1, COHb levels critically depended on both the number of cigarettes and the time of exposure to cigarette smoke. The decay of COHb in the blood of the rats was also evaluated after they had smoked three cigarettes for 1 h and after they were returned to the normal environment. As shown in Fig. 1 the COHb reached the maximum at the end of the smoking session but in about 2 h returned to pre-smoking values. The evaluation of the kinetics of alveolar and peritoneal macrophages was carried out in rats that, after undergoing smoking (three cigarettes within a hour), showed a COHb increase up to 16.4%. Figure 2 (panel A) indicates that the total number of macrophages

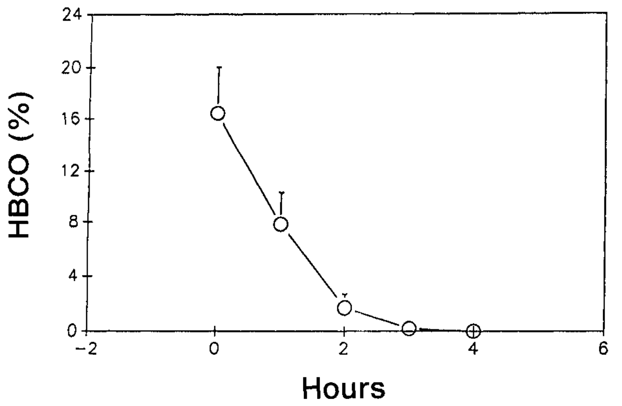

FIG. 1. Kinetics of COHb in the blood of rats during and after a smoking session (three cigarettes for 1 h). Arrows indicate the beginning and the end of the smoking session.

present in the bronchoalveolar lavage significantly increased 8 h after smoking ($U \leq 0.05$) in respect to control rats (Fig. 2, panel B). On the other hand the number of peritoneal macrophages remained practically constant in both smokers and controls (Fig. 2, panels A and B). An increase of polymorphonuclear cells (PMN) in the broncho-alveolar lavage was also observed at 8 and 24 h after an acute smoking episode but it was not statistically significant (data not shown).

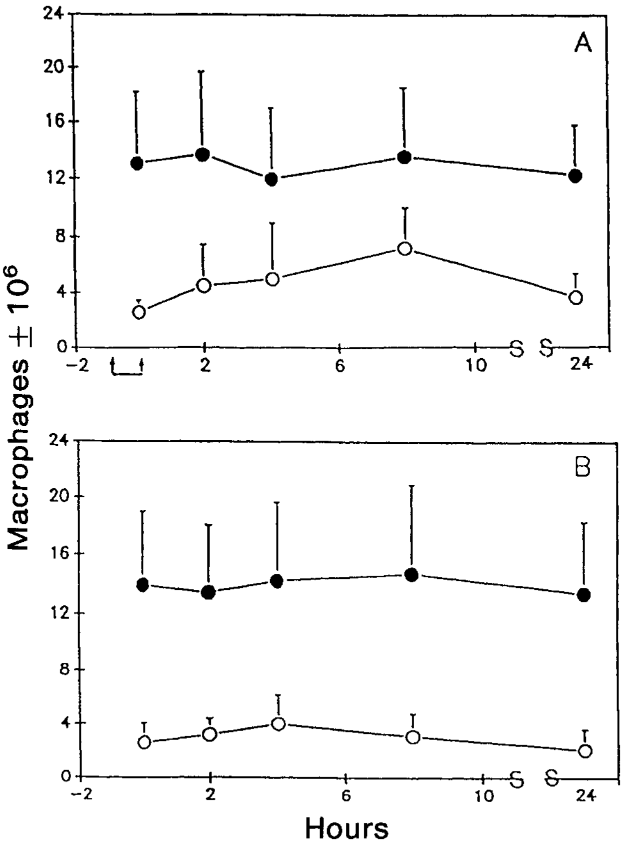

FIG. 2. Kinetics of alveolar (○) and peritoneal (●) macrophages in rats after a smoking session of three cigarettes for 1 h (panel A) and in controls (panel B). Arrows indicate the beginning and the end of the smoking session.

**Table 2.** TNFα (U/mg protein) expressed as mean ± S.D. released by alveolar macrophages collected various times after the smoking session and incubated for 24, 48 and 72 h at 37°C

| Time after smoking (h) | Incubation at 37°C (h) | | |
|---|---|---|---|
| | 24 | 48 | 72 |
| 0 | 88 ± 32 | 90 ± 40 | 76 ± 31 |
| 4 | 310 ± 151 | 283 ± 184 | 320 ± 150 |
| 8 | 525 ± 210 | 455 ± 210 | 465 ± 215 |
| 24 | 235 ± 106 | – | 250 ± 150 |
| Controls | <10 | <10 | <10 |

The appraisal of the kinetics of alveolar macrophages after acute smoking compelled us to evaluate the eventual activity of these cells by measuring the release of TNFα. As shown in Table 2, alveolar macrophages, collected at various times after acute smoking and incubated for 24, 48 and 72 h at 37°C in a humidified atmosphere, spontaneously released variable amounts of TNFα, which were higher 8 h after the smoking session. Moreover TNFα production increased up to 24 h and varied little in the following days of incubation (Table 2). Neither alveolar macrophages of air-sham exposed rats, nor peritoneal macrophages of both control and smoking rats were able to release TNFα, which was also absent either in the supernatants of BAL and PL or in plasma. When alveolar macrophages of control and smoking rats, collected 0, 8 and 24 h after the smoking session and incubated for 24 h at 37°C, were challenged with LPS, they released much more TNFα (Fig. 3) but, in such a case, the TNFα release was significantly lower for the smoking group with respect to the control rats.

### Discussion

These studies provide data on the short-term effects of acute cigarette smoking in rats. It is known that cigarette smoking in rabbits and in man causes a

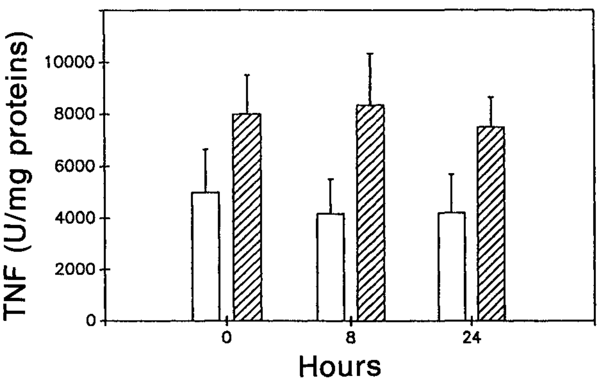

FIG. 3. TNF units/mg proteins expressed as mean ± S.D. released after induction with LPS by alveolar macrophages collected from smoker (empty columns) and control rats (narrow right diagonal columns) 0, 8 and 24 h after the smoking session.

rapid increase of COHb[8–13] and on this basis a wide range of conditions has been explored yielding a COHb increase from −1 up to 17% in rats. COHb is a reliable marker and the interanimal variability appears small. In the present experimental conditions, reproducibility among different experiments is within 10%. In order to be sure of the acute effect, we selected the protocol of smoke exposure of three cigarettes within an hour for the experiments evaluating the active state of alveolar macrophages. This gave COHb levels of about 16%, returning to pre-smoke values 2 h after the smoking session. The evaluation of the kinetic of alveolar macrophages collected from BAL after the smoking session, indicated a significant increase 8 h after the smoke episode and a little increase of PMN probably due to chemotactic factors[14,15] At the same time alveolar macrophages, incubated at 37°C in a humidified atmosphere, spontaneously released TNFα. Although TNFα release by alveolar macrophages was observed either in rats with silicosis[16] and mice infected with influenza virus,[17] or in man as a consequence of Sepsis Syndrome[18] and Adult Respiratory Distress Syndrome,[19] our results demonstrated for the first time that alveolar macrophages, collected after an acute smoking episode, were in an active state and spontaneously released TNFα. The absence of this cytokine in the BAL could be due to both the dilution and the short time for the cell collection. Additionally, the absence of TNFα in plasma could be explained by both the dilution in body fluids and the rapid clearance of this substance.[20,21] At a later date the eventual local catabolism of TNFα will be reported. Peritoneal macrophages, representing a resident population remote from the primary site of smoke and perhaps not having direct contact with smoke pollutants and products, were unable to spontaneously release TNFα. This clearly indicates that these cells were in a resting state. However, differences in regulation of TNFα production by either human,[22] or murine[23] alveolar macrophages stimulated with LPS are well known.

Finally we wanted to ascertain whether a classical inducer such as LPS antagonizes or synergizes with the effect of smoke on alveolar macrophages. It was found that alveolar macrophages collected from control rats released much more TNFα than those collected from smokers, probably because of a negative feed-back and enhanced breakdown due to proteinases released during the incubation period.[24]

### References

1. Anderson R. Assessment of the roles of vitamin C, vitamin E, and β-carotene in the modulation of oxidant stress mediated by cigarette smoke-activated phagocytes. *Am J Clin Nutr* 1991; **53**: 358S–361S.
2. Djordjevic MV, Sigountos CW, Hoffmann D, *et al.* Assessment of major carcinogens and alkaloids in the tobacco and mainstream smoke of USSR cigarettes. *Int J Cancer* 1991; **47**: 348–351.
3. Robbins RA, Thompson AB, Koyama S, *et al.* Cigarette smoking and neutrophil migration. *J Immunol Res* 1990; **2**: 178–188.
4. Bonta IL, Ben-Efraim S, Mozes T, Fieren MWJA. Tumor necrosis factor in inflammation: relation to other mediators and to macrophage antitumour defence. *Pharmacol Res* 1991; **24**: 115–129.
5. Bachwich PR, Chensue SW, Larrick JW, Kunkel SW. Tumor necrosis factor stimulates interleukin-1 and prostaglandin $E_2$ production in resting macrophages. *Biochem Biophys Res Commun* 1986; **136**: 94–101.
6. Camussi G, Albano E, Tetta C, Bussolino F. The molecular action of tumor necrosis factor-α. *Eur J Biochem* 1991; **202**: 3–14.
7. Imaizumi T, Satoh K, Yoshida H, Kawamura Y, Hiramoto M, Takamatsu S. Effect of cigarette smoking on the levels of platelet-activating factor-like lipid(s) in plasma lipoproteins. *Atherosclerosis* 1991; **87**: 47–55.
8. Bosken CH, Doerschuk CM, English D, Hogg JC. Neutrophil kinetics during active cigarette smoking in rabbits. *J Appl Physiol* 1991; **71**: 630-637.
9. Minamisawa S, Komuro E, Niki E. Hemolysis of rabbit erythrocytes induced by cigarette smoke. *Life Sci* 1990; **47**: 2207–2215.
10. Brown GM, Drost E, Donaldson K, MacGregor I, MacNee W. Reduction of the proteolytic activity of neutrophils by exposure to cigarette smoke *in vitro. Exp Lung Res* 1991; **17**: 923–937.
11. Lowry OH, Rosebrough NJ, Farr AL, Randall RJ. Protein measurement with the folin phenol reagent. *J Biol Chem* 1951; **193**: 265–275.
12. Ruff MR, Gifford GE. Tumor necrosis factor. In: Pick E, ed. *Lymphokines.* New York: Academic Press, 1981; 235–272.
13. MacNee W, Wiggs B, Belzberg AS, Hogg JC. The effect of cigarette smoking on neutrophil kinetics in human lungs. *New Engl J Med* 1989; **321**: 924–928.
14. Kunkel SL, Standiford T, Kasahara K, Strieter RM. Interleukin-8 (IL-8): the major neutrophil chemotactic factor in the lung. *Exp Lung Res* 1991; **17**: 17–23.
15. MacNee W, Selby C. Neutrophil kinetics in the lungs. *Clin Sci* 1990; **79**: 97–107.
16. Mohr C, Gemsa D, Graebner C, *et al.* Systemic macrophage stimulation in rats with silicosis: enhanced release of tumor necrosis factor-α from alveolar and peritoneal macrophages. *Am J Respir Cell Mol Biol* 1991; **5**: 395–402.
17. Vacheron F, Rudent A, Perin S, Labarre C, Quero AM, Guenounou M. Production of interleukin 1 and tumour necrosis factor activities in bronchoalveolar washings following infection of mice by influenza virus. *J Gen Virol* 1990; **71**: 477–479.
18. Kelley J. Role of cytokines in lung diseases. *EOS-J Immunol Immunopharmacol* 1991; **XI**: 140-144.
19. Hyers TM, Tricomi SM, Dettenmeier PA, Fowler AA. Tumor necrosis factor levels in serum and bronchoalveolar lavage fluid of patients with the adult respiratory distress syndrome. *Am Rev Respir Dis* 1991; **144**: 268–271.
20. Bocci V, Pacini A, Pessina GP, Maioli E, Naldini A. Studies on tumor necrosis factor (TNF). I. Pharmacokinetics of Human Recombinant TNF in rabbits and monkeys after intravenous administration. *Gen Pharmacol* 1987; **18**: 343–346.
21. Pessina GP, Pacini A, Bocci V, Maioli E, Naldini A. Studies on tumor necrosis factor (TNF): II. Metabolic fate and distribution of human recombinant TNF. *Lymphokine Res* 1987; **6**: 35–44.
22. Becker S, Devlin RB, Haskill JS. Differential production of tumor necrosis factor, macrophage colony stimulating factor, and interleukin-1 by human alveolar macrophages. *J Leuk Biol* 1989; **45**: 353–361.
23. Stein M, Gordon S. Regulation of tumor necrosis factor (TNF) release by murine peritoneal macrophages: role of cell stimulation and specific phagocytic plasma membrane receptors. *Eur J Immunol* 1991; **21**: 431–437.
24. Ogushi F, Hubbard RC, Vogelmeier C, Fells GA, Crystal RG. Risk factors for emphysema. Cigarette smoking is associated with a reduction in the association rate constant of lung α1-antitrypsin for neutrophil elastase. *J Clin Invest* 1991; **87**: 1060–1065.

ACKNOWLEDGEMENTS. This work was supported by contract no 91.00089 PF41 Targeted Project "Prevention and Control Disease Factors", Subproject 02, CNR-Roma.

**Received 29 December 1992; accepted in revised form 18 January 1993**
